# Interoptic, Trans-lamina Terminalis, Opticocarotid Triangle, and Caroticosylvian Windows From Mini-Supraorbital, Frontomedial, and Pterional Perspectives: A Comparative Cadaver Study With Artificial Lesions

**DOI:** 10.3389/fsurg.2019.00040

**Published:** 2019-07-16

**Authors:** Sasan Darius Adib, Stephan Herlan, Florian H. Ebner, Bernhard Hirt, Marcos Tatagiba, Juergen Honegger

**Affiliations:** ^1^Department of Neurosurgery, University of Tübingen, Tübingen, Germany; ^2^Department of Clinical Anatomy and Cell Analysis, University of Tübingen, Tübingen, Germany

**Keywords:** interoptic window, trans-lamina terminalis approach, opticocarotid triangle window, caroticosylvian window, artificial lesions

## Abstract

**Introduction:** The mini-supraorbital (MSO) and pterional (PT) approaches have been compared in a number of studies focusing on the treatment of aneurysms, craniopharyngiomas, and meningiomas. The goal of this study was to analyze the surgical exposure to different artificial lesions through interoptic (IO), trans-lamina terminalis (TLT), opticocarotid triangle (OCT), and caroticosylvian (CS) windows from the MSO, frontomedial (FM), and PT perspectives.

**Methods:** The MSO, PT, and FM approaches were performed sequentially in two fixed cadaver heads. Three colored spheres were placed around the optic chiasm: (1) between the optic nerves; (2) between the optic nerve and the internal carotid artery; and (3) between the internal carotid artery and the oculomotor nerve. The surgical exposures to these structures by using the IO, TLT, OCT, and CS windows were compared.

**Results:** (1) IO window: from the MSO and PT approaches, the total surgical exposure mainly allows visualization of contralateral lesions. The FM approach was superior for exploration of both sides of the area between the optic nerves. (2) TLT pathway: the MSO and PT approaches mainly expose the contralateral third ventricle wall. (3) OCT window: the PT approach allows exposure of a larger part of the sphere between the optic nerve and the internal carotid artery than the MSO approach. (4) CS window: the PT approach allows a better exposure of lateral structures such as the oculomotor nerve and of the medial prepontine area in comparison to the MSO approach.

**Conclusion:** Simulation of the surgical situation with artificial lesions is a good model for comparing surgical perspectives and for analyzing feasibility of lesion exposure and resection.

## Introduction

Mini-supraorbital (MSO) approach (also known as supraorbital keyhole approach) (1–3) and pterional (PT) approach (4) have been compared in a number of studies focusing on the treatment of aneurysms (5–7), craniopharyngiomas (8–10), and meningiomas (11–13), and they have been described in terms of their variants (14) and advantages (13, 15–17). For the surgical management of these pathologies, there is a huge controversy regarding which approach should be preferred.

Figueiredo et al. (1) reported that there were no statistical differences in the total area of surgical exposure of six defined points when they compared the MSO and PT approaches, but there was a difference in the horizontal and vertical working angles. Cheng et al. (18) reported similar results and found that the area of exposure of the parasellar region using the smaller supraorbital keyhole approach is as adequate as the larger supraorbital and PT approaches. In contrast to the anatomical studies by Figueiredo et al. (1) and Cheng et al. (18), Kang et al. (6) found that when analyzing postoperative contrast-enhanced brain computed tomographic scans and measuring the areas of exposure, even the mini-PT keyhole craniotomy (MPKC) had a larger mean area of exposure than that of the MSO and also provided a significantly larger range of microscopically operable working angles for the anterior communicating artery (ACoA), as well as bifurcation of the middle cerebral artery and internal carotid artery (ICA) (6), within a short distance (6).

Artificial lesions in a cadaver study for the analysis of the surgical exposure of the skull base and other structures have been described in single studies (19, 20). The goal of this study was to analyze the exposure to different artificial lesions from MSO, frontomedial (FM), and PT approaches by using various corridors of exposing the area under and around the optic chiasm: (1) interoptic (IO), (2) trans-lamina terminalis (TLT), (3) opticocarotid triangle (OCT), and (4) caroticosylvian (CS) windows.

## Method

The MSO, FM, and PT approaches were performed in two silicon-injected (Wacker Chemie AG, Munich, Germany) ETOH (SAV Liquid Production GmbH, Flintsbach am Inn, Germany)-fixed specimens obtained from the donor program of the Anatomical Institute in a stepwise manner. The anatomical study was approved by the ethics committee (237/2007BO).

The neurosurgical dissections were performed in the anatomical lab using standard neurosurgical equipment (Aesculap, Tuttlingen, Germany), a Mayfield headholder (Schaerer Medical AG, Münsingen, Switzerland), and a triumph operating table (TRUMPF Medizin Systeme GmbH + Co., Saalfeld, Germany) to simulate realistic surgical conditions. A standard operating microscope (OPMI, Carl Zeiss Company, Oberkochen, Germany) was used.

**Figure 1:**
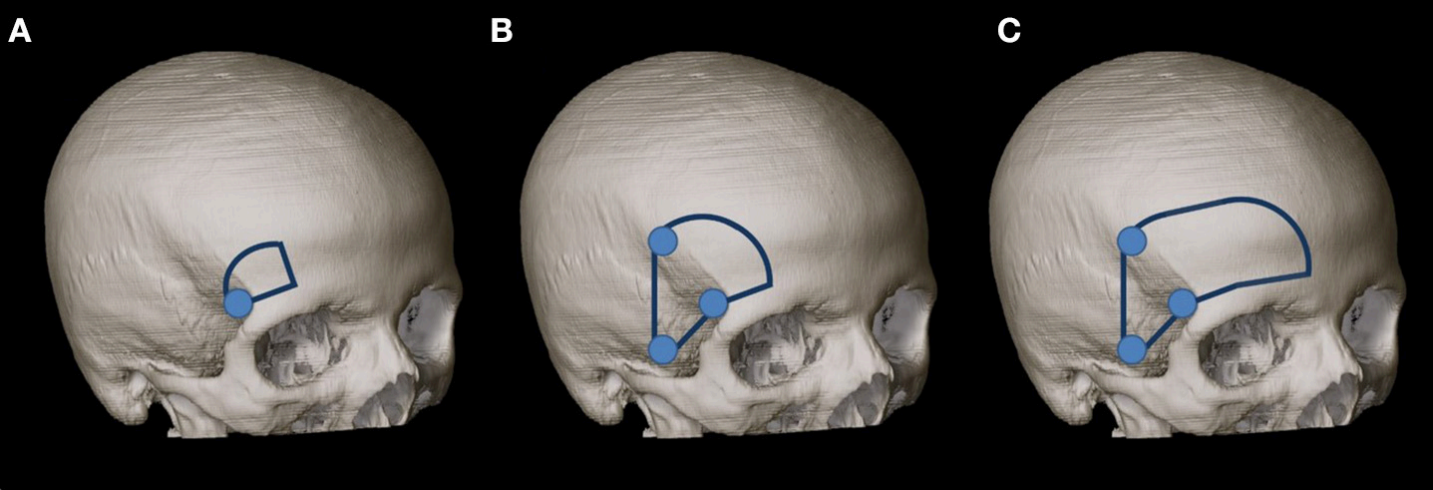
**(A)** MSO craniotomy was performed according to Reisch et al. (2) with a 2 × 2.5-cm extension. **(B)** Second, the MSO approach was extended to a PT approach according to Yaşargil et al. (21) with a size of 4 × 6 cm. **(C)** The craniotomy was enlarged (as our third step) for the exposure to the frontal midline.

The dissection was performed stepwise using a standard skin incision from the midline to the zygoma and the interfascial dissection technique described by Yaşargil et al. (21). First, the MSO craniotomy was performed according to Reisch et al. (2) with a 2 × 2.5-cm extension (Figure 1A), and the surgical exposure of the neurovascular structures around the optic chiasm was analyzed from the perspective of the MSO approach. Structures exposed included the planum sphenoidale, tuberculum sellae, anterior clinoid process on both sides, lamina terminalis, cranial nerves (CNs) CN I, CN II (including the cranial surface of the optic chiasm), and CN III ipsilaterally and contralaterally, and, finally, arteries from the Circle of Willis including the ICA, first and second segments of the anterior (A1 and A2), middle (M1 and M2), and posterior (P1 and P2) cerebral arteries, posterior communicating artery (PCOA) on both sides, and distal basilar artery (BA).

Second, the MSO approach was extended to a PT approach according to Yaşargil et al. (21) with a size of 4 × 6 cm (Figure 1B). The lateral sphenoid ridge was drilled, and the sphenoidal compartment of the sylvian fissure was opened, as described by Figueiredo et al. (22). The surgical exposure was analyzed with regard to the above-mentioned structures.

**Figure 2:**
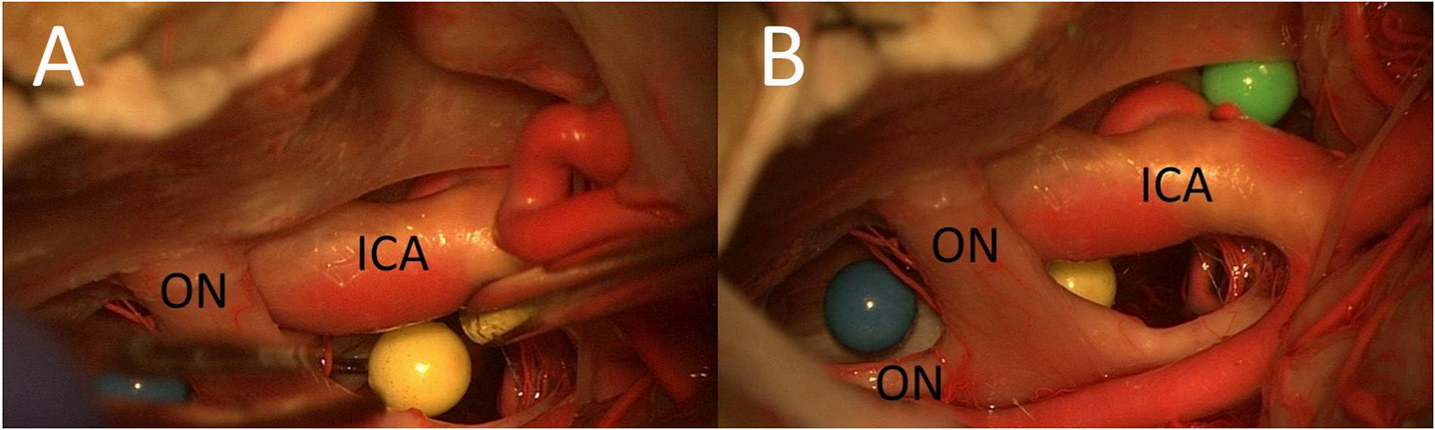
Placement of artificial lesions (three colored spheres) in key locations **(A,B)** (ON, optic nerve).

Three colored spheres (4-mm diameter) were placed around the optic chiasm in key locations (Figure 2) to analyze the corridors around the optic chiasm. The radius of the spheres was 2 mm; this corresponds to a volume of 33.51 mm^3^.

The key locations were as follows:

1. Between the optic nerves over the sellar diaphragm (blue) (in both cases, we had a midfixed optic chiasm)
2. Between the right optic nerve and the right ICA (yellow)
3. Lateral to the ICA and medial to the oculomotor nerve on the sphenoid wing (green).

In the second cadaver, the yellow and the green spheres were placed in the opposite way, which helped differentiate the photos from those of the first cadaver.

Furthermore, the lamina terminalis was opened [according to Maira et al. (23)] to access the third ventricle.

**Figure 3:**
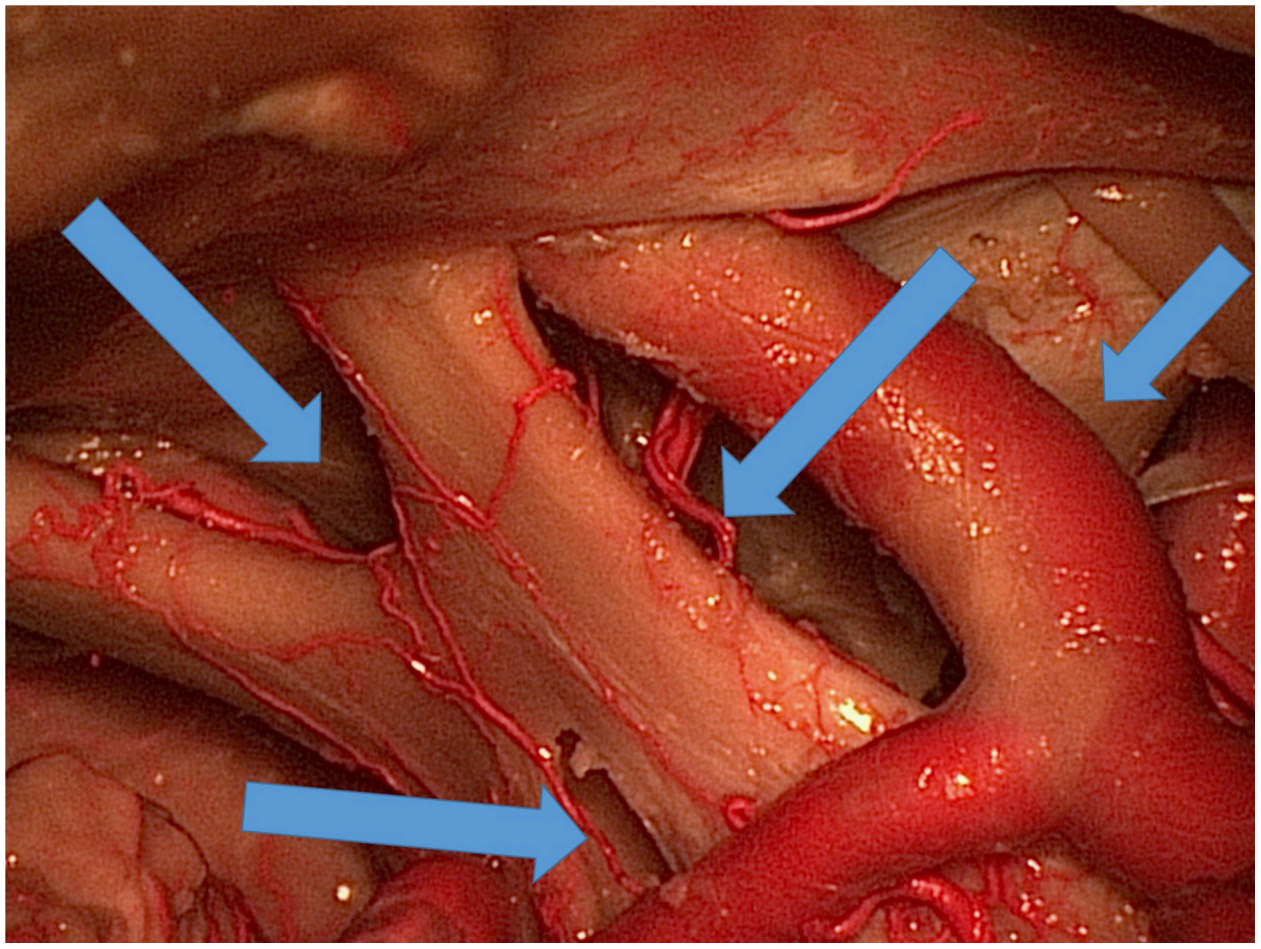
Various ways (blue arrows) of exposing the area under and around the optic chiasm and through the lamina terminalis to the third ventricle.

Four different surgical corridors were used to explore the three colored spheres, third ventricle, area under the optic chiasm, and prepontine area (Figure 3): the IO, TLT, OCT, and CS corridors.

The surgical exposures of the artificial lesions using the four corridors were compared between the MSO and PT approaches. The bony flaps were alternatingly refixed and removed to allow direct comparison of the MSO and PT approaches. The two approaches were compared with regard to the surgical access to the spheres (from the maximal lateral and medial working angles) and the surgical exposure of the third ventricle (through the lamina terminalis).

Because of the limited exposure of the IO area (using the IO window) and the third ventricle (using the TLT corridor) from the MSO and PT views, we enlarged the craniotomy (as our third step) for the exposure to the frontal midline (Figure 1C). The exposure of the third ventricle through the TLT corridor and the areas between the optic nerves was compared between the above-mentioned craniotomies and the FM approach.

## Results

### Surgical Exposure of Optic Chiasm and Circulus Arteriosus

The MSO and PT approaches allowed in both cadavers a good exposure of the planum sphenoidale, tuberculum sellae, anterior clinoid process, as well as optic chiasm and lamina terminalis.

Furthermore, it was possible to reach CN I, CN II, and CN III ipsilaterally and (with dissection of arachnoid adhesions) contralaterally as well as the cranial surface of the optic chiasm. The MSO and PT approaches allowed visualization of multiple arteries of the circulus arteriosus, including the ICA, A1, A2, M1, M2, P1, P2, PCOA on both sides, and distal BA.

### Surgical Corridors

#### IO Window

**Figure 4:**
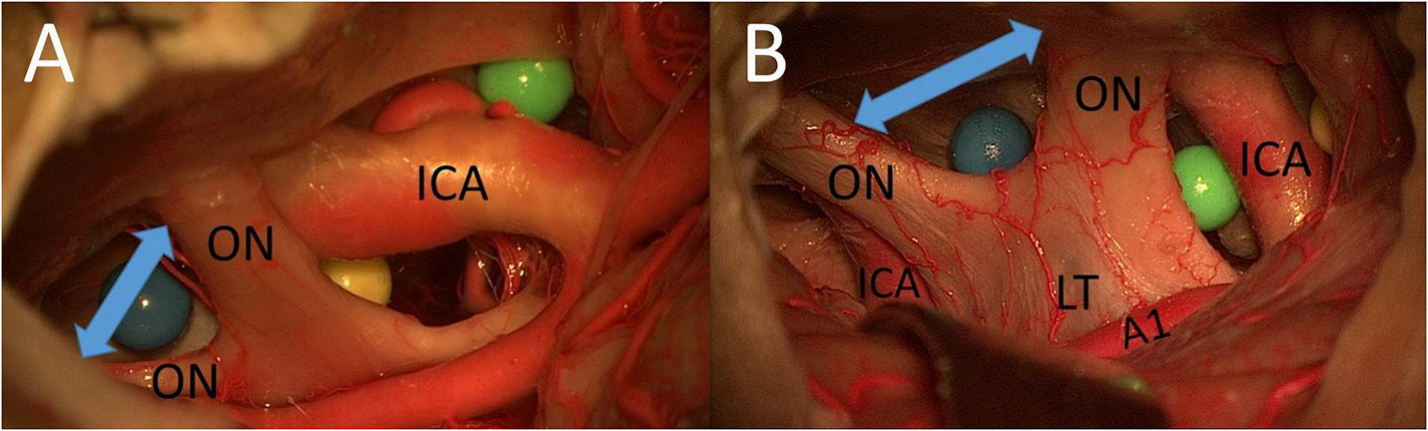
**(A)** Cadaver 1: IO window from PT perspective. **(B)** Cadaver 2: IO window from unilateral FM approach (ON, optic nerve; A1, proximal segment of anterior cerebral artery; LT, lamina terminalis).

In both cadavers, we had a midfixed optic chiasm, which means that it was possible to use the IO window as a surgical pathway (without drilling the limbus sphenoidale). Using the MSO and PT approaches, the total area of surgical exposure between the two optic nerves to the sellar diaphragm was smaller than that using a unilateral FM approach and allowed access mainly to contralateral lesions. The FM approach was superior for lesions between the two optic nerves with extension to both sides (Figure 4).

#### TLT Approach

**Figure 5:**
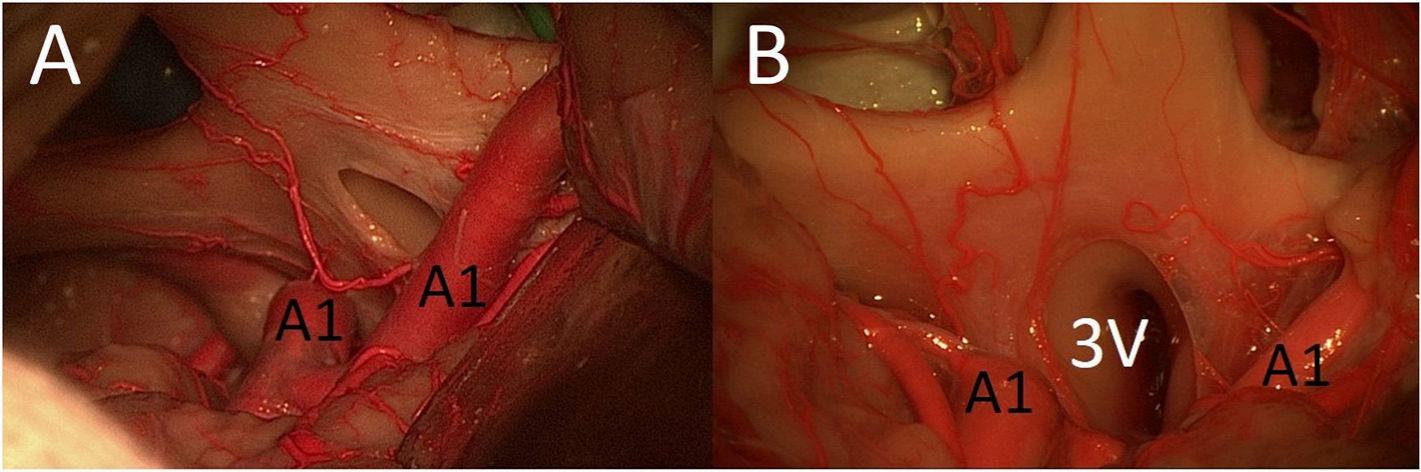
**(A)** MSO and PT approaches allowed access to contralateral lesions of the third ventricle through the lamina terminalis. **(B)** The FM approach was the best way to reach deep lesions of the third ventricle through the lamina terminalis (3V, third ventricle; A1, proximal segment of anterior cerebral artery; LT, lamina terminalis).

After opening the LT, the MSO and PT approaches allowed access to contralateral lesions of the third ventricle through the lamina terminalis (Figure 5). The (unilateral or bilateral) FM approach was the best way to reach deep lesions of the third ventricle through the LT using a direct approach (with straight perspective) and to expose the floor and both sides of the third ventricle (Figure 5). The FM approach even allowed the opening of the floor of the third ventricle and exposed the prepontine area and BA.

#### OCT Window

**Figure 6:**
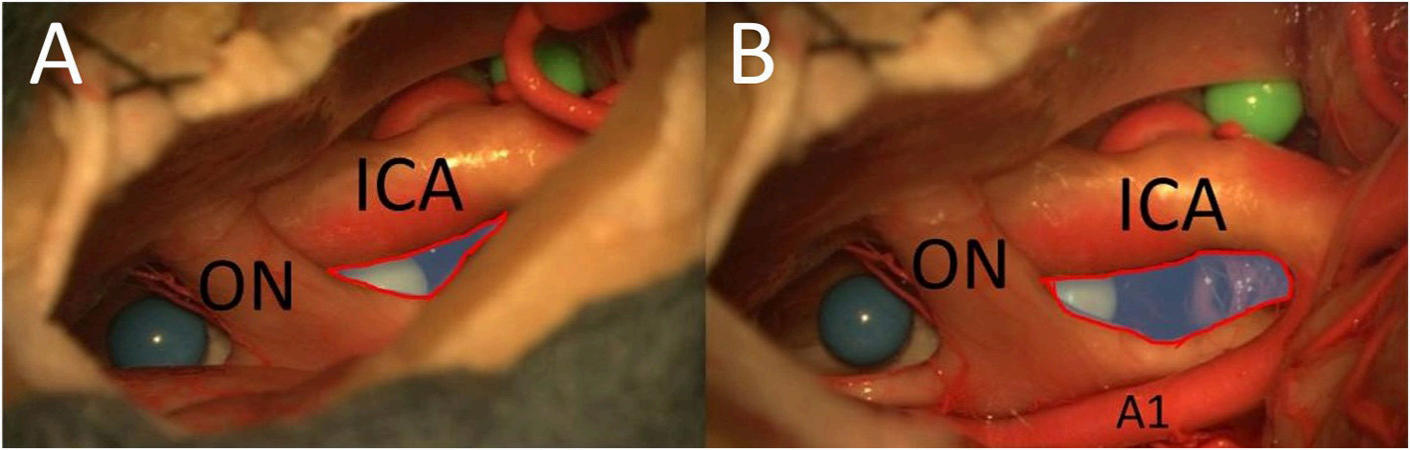
The opticocarotid window from MSO **(A)** and PT approaches **(B)** (blue area with red frame = area of exposure of the OCT) (ON, optic nerve; A1, proximal segment of anterior cerebral artery).

**Figure 7:**
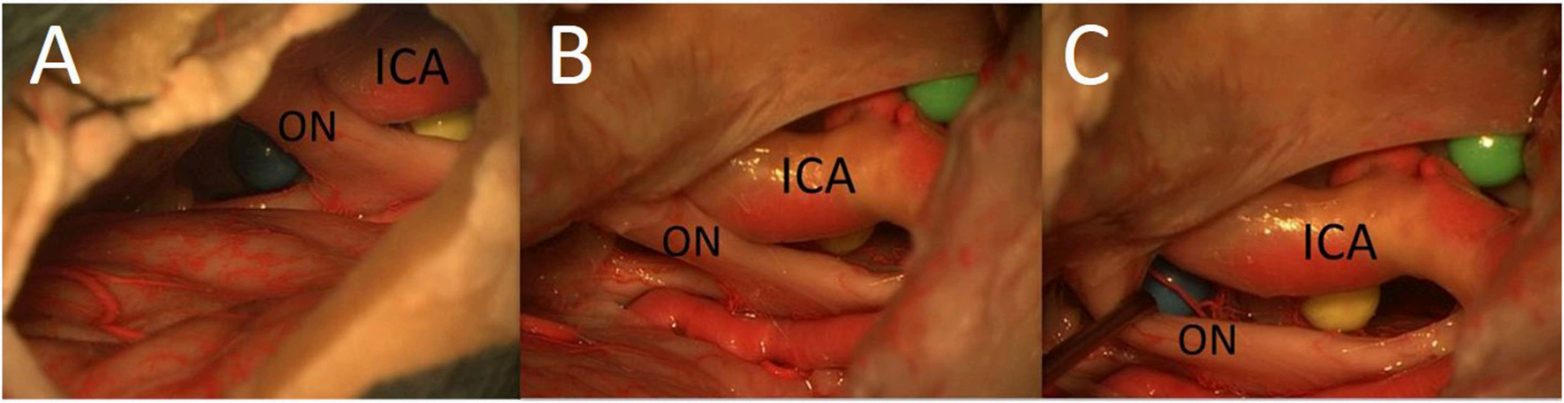
The area under the optic chiasm: it is not possible to reach the area under the optic chiasm from the lateral aspect using the MSO approach **(A)**; the area under the optic chiasm with the undersurface of the chiasm is easily visualized from lateral perspective through the opticocarotid window using the PT approach **(B,C)** (ON, optic nerve).

The PT approach allowed exposure of a larger part of the surface of the sphere (especially also of the posterior part) between the right optic nerve and the right carotid artery compared with the MSO approach because of the greater vertical working angle and shorter distance (Figure 6). Because of the greater vertical working angle and lateral view, it was also possible to reach the area under the optic chiasm (including the blue sphere and pituitary stalk) from the lateral aspect through the OCT window using the PT approach (Figure 7), which was not possible using the MSO approach. The larger vertical angle and the improved lateral view of the PT approach even allowed access to structures on the contralateral side and to the upper prepontine area. Mobilization of the ICA enlarged the OCT window and the surgical exposure of the structures.

#### CS Approach/Carotid-Oculomotor Window

**Figure 8:**
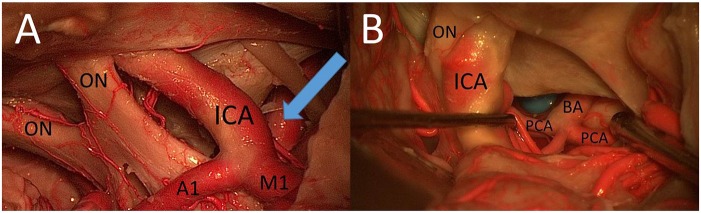
**(A)** CS window from PT perspective. **(B)** The prepontine area through the caroticosylvian window using the PT approach: mobilization of the ICA was necessary to increase the view to the BA, the PCA, the rami ad pontem, and the anterior upper surface of the brainstem (ON, optic nerve; A1, proximal segment of anterior cerebral artery; M1, proximal segment of middle cerebral artery).

The PT approach allowed better exposure of lateral structures such as the oculomotor nerve (Figure 8) and the sphere lateral to the ICA in comparison with the MSO approach, mainly because of a more lateral view but also because of a larger vertical working angle.

#### Surgical Exposure of the Basilar Apex Through the Opticocarotid and CS Approach

With the use of the PT approach and the view through the opticocarotid and/or CS window, it was possible to reach the prepontine area, including the apex of the BA, posterior cerebral artery (PCA), rami ad pontem, and anterior upper surface of the brainstem (Figure 8).

Mobilization of the ICA was in both cases necessary to increase the view of this area. The apex of the BA was also reachable using the MSO approach through the OCT and CS windows, but the view was more limited and it was not possible in our two cadavers to visualize the rami ad pontem using this approach.

## Discussion

### The Surgical Exposure of Neurovascular Structures From the MSO and PT Approaches

In the first part of our study, we compared the surgical exposure of neurovascular structures around the optic chiasm. Similar to the results of Figueiredo et al. (1) and Perneczky et al. (24), we found that both MSO and PT allow good surgical exposure of the planum sphenoidale, tuberculum sellae, anterior clinoid process on both sides, optic chiasm, lamina terminalis, cranial nerves (CN I–III), the ICA, A1, A2, M1, M2, P1, P2, and PCOA, and the distal BA from the circulus arteriosus.

### The Perspective to Artificial Lesions Through Anatomical Windows

Yaşargil et al. (25) described different anatomical windows that had been used as pathways by using a PT-transsylvian approach and referred to them as prechiasmatic, opticocarotid, carotidotentorial triangle, and lamina terminalis pathways. In addition, other authors defined similar windows (Table 1) (26–30).

**Table 1:** Windows around the optic chiasm: the window between the optic nerves is mostly addressed as prechiasmatic (25, 26) or IO (27) window and the window lateral to the carotid artery as the carotidosylvian (27), carotidotemporal (28), retrocarotid (29), or carotidotentorial (25) window.

**References**	**Window 1**	**Window 2**	**Window 3**	**Window 4**	**Window 5**
Yaşargil et al. (25)	Prechiasmatic	Opticocarotid	Carotidotentorial	Lamina terminalis	x
Koos et al. (27)	interoptic	Opticocarotid	Carotidosylvian	Lamina terminalis	x
Tanaka et al. (29)	x	Opticocarotid	Retrocarotid (medial, lateral)	x	x
Honegger and Tatagiba (26)	Prechiasmatic	Opticocarotid	Lateral to carotid artery	Lamina terminalis	Tuberculum sellae
Metwali et al. (28)	Subchiasmatic	Opticocarotid	Carotidotemporal	x	x
Cikla et al. (30)	Subchiasmatic	Opticocarotid	x	Lamina terminalis	x

*Tanaka et al. (29) subclassified the retrocarotid window in the medial retrocarotid space between the ICA and the PCOA, and the lateral retrocarotid space between the PCOA and the oculomotor nerve, which might be used for the surgical management of BA bifurcation aneurysms*.

Different strategies are further described in the literature to enlarge the windows and to expose the different structures. These include drilling the limbus sphenoidale, drilling the lateral part of the tuberculum sellae, performing an anterior and posterior clinoidectomy, mobilizing the ICA (31), drilling the dorsum sellae (31), dividing the PCoA from the posterior third portion of the vessel (31), and, in the case of the PT approach, enlarging the opening of the sylvian fissure.

### IO Window/Prechiasmatic Window

#### Anatomical Parameters of the IO Window

In our study, we found that the total area of surgical exposure between the two optic nerves to both the sellar diaphragm and the blue sphere is limited using the MSO and PT approaches and, thus, enables the reach of mainly contralateral lesions. The FM approach was the superior approach for exploring both sides of the area between the optic nerves.

The possibility of using the IO window to perform a tumor resection or clip of contralateral aneurysms depends on the anatomical characteristics of the optic chiasm and the surrounding structures. Three different types of “fixation” of the optic chiasm have been described in previous studies, and the incidences of a normally fixed, prefixed, and a post-fixed chiasm are ~70–80, 5–15, and 15–17%, respectively (32–35). In both of our cases, the optic chiasm was fixed normally, and it was possible to use the IO window as a surgical corridor.

### Trans-lamina Terminals Pathway

The fenestration of the LT is an often-used and save (36) approach to lesions of the third ventricle (4, 23, 37, 38). Our study shows that both the MSO and the PT approaches allow access only to reach contralateral lesions of the third ventricle through the lamina terminalis. Thus, the FM approach would be the best way to reach deep lesions of the third ventricle. Furthermore, it offers a straight perspective and allows visualization of both sides and of the floor of the third ventricle.

Several approaches have been used to expose the third ventricle via the LT approach. Tubbs et al. (35) separated these using anterior midline approaches (like subfrontal and interhemispheric), which offer a frontal trajectory of the LT and the third ventricle, “providing a wide surgical corridor to view the lateral walls of the hypothalamus and the third ventricle” and anterolateral (e.g., PT and orbitozygomatic), which offer a shorter distance to the suprasellar region (35).

Liu et al. (39) mentioned that they used the “translamina terminalis corridor via the transbasal subfrontal approach” for the management of craniopharyngiomas and concluded that “the surgical view of the optic chiasm is oblique and prevents adequate visualization of the ipsilateral wall of the third ventricle.” These results are similar to our results.

### OCT Window

In our study, we showed that the PT approach allows the exposure of a larger part of the surface of the sphere between the right optic nerve and the right carotid artery than does the MSO approach, especially the posterior part of the sphere. The reason is a perspective from more below and lateral with the PT approach. Furthermore, the PT approach also made it possible to reach the area under the optic chiasm through the OCT window, which cannot be visualized with an MSO approach.

Yasargil et al. (40) and Nagasawa et al. (41) concluded the window between the optic nerve, the ICA, and the anterior cerebral artery may be used when a 5–10 mm space exists between them (40).

Evans et al. (42) investigated the use of anterior clinoidectomy and showed that the exposure of the width and the length of the OCT and of the suprasellar and periclinoid regions improves dramatically, and Matsuyama et al. (43) studied the value of mobilization of the ICA and showed that it is an efficient procedure to widen the surgical corridor of the OCT.

#### The OCT Window as Access to the Prepontine Area

In our study, we demonstrated that the PT approach even enables the surgeon to reach the upper prepontine area. Mobilization of the ICA enlarges the OCT window and the surgical exposure of these structures. The prepontine area and the BA were also accessible from the MSO approach, but in most cases, only the upper part could be reached.

### Carotidosylvian/Retrocarotid Window

The carotidosylvian (or retrocarotid) window has also been described by Yaşargil et al. (25). Tanaka et al. (29) subclassified it as the medial and the lateral retrocarotid window in relation to the PCOA. In their study, they showed the use of the medial and lateral retrocarotid window for the surgical treatment of BA bifurcation aneurysms. In seven cases, they resected the posterior clinoid to expose the proximal BA for possible temporary clipping via a retrocarotid approach.

Youssef et al. (44) also used the carotid-oculomotor window for the exposure of upper BA aneurysms and concluded that the anterior clinoidectomy and ICA mobilization enlarge the carotidosylvian window by 44% anteriorly and by 28% posteriorly, while posterior clinoidectomy enlarges the exposed length of the BA by 69% (44).

Our study showed that the PT approach allows good exposure of lateral structures (e.g., oculomotor nerve) through the carotid sylvian window as demonstrated by the excellent view of the artificial lesion lateral to the ICA. Only the edge of the sphere could be visualized when using the MSO approach.

### Limitations

A limitation of this study is the small number of specimens (two samples), which did not allow a statistical analysis and limits the analysis of the influence of variations in bone structures and neurovascular structures on the surgical exposure to different structures.

In both of our cases, the optic chiasm was fixed normally. A prefixed or a post-fixed chiasm will change the surgical exposure through the IO window. Also, other variations (such as the variants of vertebrobasilar complex, etc.) have an influence on the results.

## Conclusion

Anatomical studies usually have the limitation that in most cases the analysis is based on a cadaver without any cerebral pathology. The use artificial lesions in a cadaver study for the analysis of the surgical exposure of different approaches helps to simulate different pathologies (19, 20). This study shows that artificial lesions might also be a good instrument to analyze the perspective differences of the MSO, FM, and PT approaches through different surgical windows around the optic chiasm. Simulating the surgical situation using artificial lesions has helped us understand more clearly the differences in perspective among the various approaches.

## Data Availability

The raw data supporting the conclusions of this manuscript will be made available by the authors, without undue reservation, to any qualified researcher.

## Ethics Statement

The anatomical study was approved by the Ethics Committee (237/2007BO).

## Author Contributions

All authors listed have made substantial, direct, and intellectual contribution to the work and approved it for publication. SA performed the analyses, conceived the project, wrote the paper, and performed critical revision of the article. SH performed the analyses. FE performed the analyses and the critical revision of the article. BH performed supervision and the critical revision of the article. MT supervision and critical revision of the article. JH supervision, idea of the project, performed the analysis, and critical revision of the article.

### Conflict of Interest Statement

The authors declare that the research was conducted in the absence of any commercial or financial relationships that could be construed as a potential conflict of interest.
